# Subphrenic Abscess

**Published:** 1906-02-10

**Authors:** 


### SUBPHRENIC ABSCESS.

Although the treatment of subphrenic abscess is a purely surgical consideration, yet the diagnosis of the condition has usually to be made by the physician. In the course of a communication confessedly dealing with the surgical treatment of the lesion, Mr. Cuthbert Wallace [1] very rightly observes that "there is no class of case in the whole range of medicine and surgery where the mutual co-operation of physician and surgeon is more necessary and helpful than in subphrenic abscess. Whether the case is one in which the symptoms are primarily those of subphrenic abscess, or whether these arise secondarily to an already recognised peritoneal infection, there can be no doubt that for accurate diagnosis and efficient treatment the physician and surgeon must combine forces." The greatest difficulty occurs when there is no definite history pointing to septic peritoneal infection, for then it is necessary to distinguish primary mischief in the lung from secondary involvement due to the extension of inflammation through the diaphragm.

The causes of subphrenic abscess in Mr. Wallace's series of six cases were as follows: Appendicitis in four cases, duodenal ulcer in one case, and gastric ulcer in the remaining one. In a series of 23 cases occurring in the practice of St. Thomas's Hospital between the years 1896 and 1902 inclusive, the causes of the lesion were distributed as follows: Appendicitis, 9 cases; gastric ulcer 7 cases; doubtful, 3 cases; duodenal ulcer, 2 cases; ruptured gut, 1 case; empyema, 1 case. These figures emphasise the importance of lesions of the appendix in the causation of subphrenic abscess.

The process by which the subphrenic spaces become infected is considered by the author to depend to a great extent upon the physical configuration of the peritoneal cavity. He points out that in the supine position there are in the abdomen two watersheds, a vertical one passing down the middle line of the abdomen and represented by the median line of the omentum in front and the projection of the spine behind. The other is a transverse watershed formed by the brim of the pelvis. Therefore, fluid set free near the middle line will gravitate to the sides of the abdomen and eventually overflow into the pelvis. Conversely, if the pelvis be filled with fluid to overflowing, the excess will find its way to the loins. Infection will therefore spread to the kidney pouches in two ways; firstly, as has been explained, by a merely mechanical process, and secondly by a direct extension of the thick lymph which results from the escape of the contents of the alimentary canal consequent upon perforation. This thick lymph extends in all directions, passing to right and left until it reaches the lateral parts of the abdomen, and upwards (or downwards, according to the site of the original lesion) until it reaches the edge of the liver. Here it ascends over the upper surface of this organ, and spreads beneath the dome of the diaphragm on either side of the falciform ligament. Having reached the summit it descends again to reach an exit either by way of the spleen or kidney.

In the case of appendicitis the pus tracks upwards along the outer side of the ascending colon and passes to the upper surface of the liver by way of the kidney pouch. This process may be so slow that the primary lesion may have healed before signs of the involvement of the subphrenic space make their appearance. Occasionally there may be a direct and obvious connection between an abscess in the appendix-region and one beneath the diaphragm, this connection being either a narrow and sinuous track or a large continuous abscess cavity.

We are not here concerned with the details of the surgical treatment, but Mr. Wallace's estimate of the prognosis is valuable. "There is no doubt," he says, "that the outlook in cases where the abscess is well established is very serious if not extremely gloomy. As a rule it is possible to afford efficient drainage if there is a definite abscess cavity, but there are many cases in which the infected area is simply covered by a layer of thick and tenacious pus, and in which it is almost impossible to get rid of the infective material. In spite of all treatment the temperature remains raised and the patient emaciates. In some cases, especially those arising from appendicitis, the failure to secure satisfactory progress is due to pyæmia and septic infection of the liver: such a complication is almost invariably fatal. Secondary involvement of the pleura is serious, but by no means a fatal complication provided early drainage is established."

Of the seven cases treated by the author only two survived, both having developed the subphrenic abscess as a consequence of appendicitis. Of the 23 patients in the practice of St. Thomas's Hospital, to whom reference has already been made, 13 were males and 10 females. Of the total number 12 died and eleven were discharged recovered.

[1] Trans. Med. Soc. Lond. vol. xxviii.

